# A History of Heart Failure Is an Independent Risk Factor for Death in Patients Admitted with Coronavirus 19 Disease

**DOI:** 10.3390/jcdd8070077

**Published:** 2021-06-30

**Authors:** Francesco Castagna, Rachna Kataria, Shivank Madan, Syed Zain Ali, Karim Diab, Christopher Leyton, Angelos Arfaras-Melainis, Paul Kim, Federico M. Giorgi, Sasa Vukelic, Omar Saeed, Snehal R. Patel, Daniel B. Sims, Ulrich P. Jorde

**Affiliations:** 1 Montefiore Medical Center, Division of Cardiology, Albert Einstein College of Medicine, New York, NY 10467, USA; fcastagn@montefiore.org (F.C.); rakatari@montefiore.org (R.K.); smadan@montefiore.org (S.M.); svukelic@montefiore.org (S.V.); osaeed@montefiore.org (O.S.); snepatel@montefiore.org (S.R.P.); dsims@montefiore.org (D.B.S.); 2 Montefiore Medical Center, Department of Medicine, Albert Einstein College of Medicine, New York, NY 10467, USA; syali@montefiore.org (S.Z.A.); kdiab@montefiore.org (K.D.); cleyton@montefiore.org (C.L.); pakim@montefiore.org (P.K.); 3 Department of Medicine, NYC Health & Hospitals/Jacobi, Albert Einstein College of Medicine, New York, NY 10461, USA; angelosarfaras@gmail.com; 4 Department of Pharmacy and Biotechnology, University of Bologna, 40126 Bologna, Italy; federico.giorgi@unibo.it

**Keywords:** heart failure, cardiovascular disease, epidemiology, COVID-19, corona virus 2019, risk factor

## Abstract

Aims: The association between cardiovascular diseases, such as coronary artery disease and hypertension, and worse outcomes in COVID-19 patients has been previously demonstrated. However, the effect of a prior diagnosis of heart failure (HF) with reduced or preserved left ventricular ejection fraction on COVID-19 outcomes has not yet been established. Methods and Results: We retrospectively studied all adult patients with COVID-19 admitted to our institution from March 1st to 2nd May 2020. Patients were grouped based on the presence or absence of HF. We used competing events survival models to examine the association between HF and death, need for intubation, or need for dialysis during hospitalization. Of 4043 patients admitted with COVID-19, 335 patients (8.3%) had a prior diagnosis of HF. Patients with HF were older, had lower body mass index, and a significantly higher burden of co-morbidities compared to patients without HF, yet the two groups presented to the hospital with similar clinical severity and similar markers of systemic inflammation. Patients with HF had a higher cumulative in-hospital mortality compared to patients without HF (49.0% vs. 27.2%, *p* < 0.001) that remained statistically significant (HR = 1.383, *p* = 0.001) after adjustment for age, body mass index, and comorbidities, as well as after propensity score matching (HR = 1.528, *p* = 0.001). Notably, no differences in mortality, need for mechanical ventilation, or renal replacement therapy were observed among HF patients with preserved or reduced ejection fraction. Conclusions: The presence of HF is a risk factor of death, substantially increasing in-hospital mortality in patients admitted with COVID-19.

## 1. Introduction

In December of 2019, a series of cases of pneumonia of unknown etiology associated with exposure to a seafood market were reported from the city of Wuhan in China [1]. In January 2020, the etiological agent behind this disease was identified as a novel coronavirus named 2019-nCoV or severe acute respiratory distress syndrome coronavirus-2 (SARS-CoV-2) due to phylogenetic similarities with the previously known SARS-CoV [1]. The illness associated with this virus was labeled “Coronavirus disease 2019” (COVID-19) [1,2]. Since then, COVID-19 has spread rapidly across the globe and was declared a global pandemic by the World Health Organization on 11th March 2020. As of 15th October 2020, there are over 38 million cases reported worldwide with over 1 million fatalities [3], with almost a quarter of these in the United States alone [3]. Although different studies have shown an association between cardiovascular diseases and severity of infection or worse outcomes in COVID-19 patients [4,5,6,7], few and limited studies have specifically analyzed the effect of on heart failure (HF) [8,9]. Furthermore, no studies have considered the difference between HF with preserved ejection fraction (HFpEF) or with reduced ejection fraction (HFrEF). In the present study, we analyzed the independent association between existing HF and mortality, need for mechanical ventilation, or need for dialysis in COVID-19 patients.

## 2. Methods

### 2.1. Disclosure for Data Sharing

The data that form the basis of the presented findings are available from the corresponding author upon reasonable request.

### 2.2. Patient Selection

We retrospectively reviewed and enrolled all the patients admitted to our institution with a polymerase chain reaction (PCR)-proven diagnosis of COVID-19 obtained between 1st March 2020–2nd May 2020. Patients on mechanical circulatory support, on hemodialysis before the admission for COVID-19, and recipients of heart transplantation were excluded from the study. The study was approved by our institutional review board (Office of Human Research Affairs at Albert Einstein College of Medicine, #2020-11308). Given the retrospective nature of the study, waiver of informed consent was granted.

### 2.3. Data Collection, Exposure, and Outcomes

Demographics, clinical parameters, laboratory results, and treatments were retrieved from electronic health record system and supplemented with manual chart review. Patients were categorized into two groups, those with HF or those without HF, based on known prior history of HF and then compared for in-hospital mortality, need for intubation, and need for new dialysis during COVID-19 hospitalization. In a subgroup analysis, we compared these outcomes within the HF group classified by left ventricle ejection fraction (LVEF) as being HFrEF (LVEF ≤ 40%) or HFpEF (LVEF > 40%). The investigators had direct access to the raw data. 

### 2.4. Statistical Analysis

Continuous data are displayed as mean ± standard deviation or median {25–75% interquartile range} and compared using the Student’s *t*-test or Wilcoxon ranks-sum test, as appropriate. Categorical data are presented as percent and compared using the chi-squared test. As hospital discharge is a competing event with in-hospital mortality, need for intubation, or need for new dialysis, we used the Fine and Gray’s method of competing events regression analysis [10,11,12] to estimate the difference in the cumulative incidence of the outcomes of interest in the HF and non-HF groups. The date of 26th June 2020 was the end of the censoring period. Propensity score (PS) matching was also used to examine the association between HF and the different outcomes. HF patients were matched to non-HF patients using a 1:1 greedy matching algorithm, with a caliper width of 0.1 SD. As the purpose of this study was to identify elements in the past medical history that may affect outcomes, the choice of covariates for the PS model was based on the presence of statistically different incidences of comorbidities between the HF and non-HF patients. The chosen covariates included: history of hypertension (HTN), coronary artery disease (CAD), asthma or chronic obstructive pulmonary disease (COPD), and diabetes mellitus (DM). Additionally, age and body mass index (BMI) were also included in the model, due to known associations between these parameters and COVID-19 mortality [13]. A *p* < 0.05 was considered statistically significant. Statistical analysis was performed with SPSS (IBM Corp, ver. 25, Armonk, NY, USA) and the R v3.3 package cmprsk (R Foundation for Statistical Computing, ver 3.3, Vienna, Austria) 

## 3. Results

### 3.1. Demographics and Comorbilities

We identified 4043 patients admitted with COVID-19 during the study period. Data are presented in Table 1. The number of 335 patients (8.3%) had a past medical history of HF. Of these, 114 (2.8%) had HFrEF. Compared to patients without HF, patients with HF were older (73 {65–82} vs. 65 {54–76} years, *p* < 0.001), had lower body mass index (BMI) (27.9 {24.1–32.9} vs. 28.8 {24.9–33.3} kg/m^2^, *p* = 0.063), and had a significantly higher burden of co-morbidities: diabetes was present in 73.7% patients with HF vs. 49.3% of patients without HF, hypertension in 91.9% vs. 69.5%, CAD in 74.3% vs. 19.5%, and COPD/Asthma 52.5% vs. 25.3% (all *p* < 0.001) (Table 1).

### 3.2. Patient Characteristics at Presentation

Diastolic blood pressure and heart rate were slightly lower in patients with HF compared to patients without HF: 73 (60–82) vs. 75 (65–84) mmHg, *p* = 0.001 and 92 (78–106) vs. 99 (87–113) beats per minute, *p* < 0.001, respectively. We observed a statistical, but not clinically meaningful, difference in the temperature at arrival between patients with HF and patients without HF (98.7 {97.9–99.9} vs. 98.9 {98.2–100.1} F, *p* = 0.002), as well as in the pulse oximeter saturation (95 {91–98} vs. 95 {90–98} %, *p* = 0.047). Respiratory rate at presentation (20 {18–24} vs. 20 {18–22} breath per minute, *p* = 0.274) was similar between the two groups. Patients with HF had a worse baseline estimated glomerular filtration rate (EGFR) (40.7 {22.8–59.2} vs. 66.0 {40.5–89} mL/min/1.73 m^2^, *p* < 0.001) and lower hemoglobin concentration (11.9 {10.2–13.6} vs. 13 {11.6–14.3} g/dL, *p* < 0.001), when compared to patients without HF. Both groups had similar elevations of lactic acid (2.3 {1.6–3.2} vs. 2.1 {1.6–3.0} mmol/L, *p* = 0.161). Markers of inflammation did not differ between the two groups: C-reactive protein (10 {4.1–21.8} vs. 10.6 {4.4–18.4} µg/mL, *p* = 0.759), lactate dehydrogenase (392 {284–562} vs. 401 {294–549} U/L, *p* = 0.846), and ferritin (615 {323–1155} vs. 739 {357–1472} ng/mL, *p* = 0.065). However, compared to patients without HF, patients with HF had a higher pro-BNP (2727 {1068–9108} vs. 306 {90–1096} pg/mL, *p* < 0.001), troponin T (0.03 {0.01–0.07} vs. 0.01 {0.01–0.02} ng/mL, *p* < 0.001), and D-dimer (2.5 {1.1–5.4} vs. 1.7 {0.9–4} µg/mL, *p* = 0.024). Interestingly, creatine kinase was lower in patients with HF (151 {76–342} vs. 182 {90–451} U/L, *p* = 0.008). Baseline characteristics of patients with HF classified by LVEF (≤40% vs. >40%) are shown in Supplemental Table S1.

### 3.3. Therapies in House

A larger proportion of patients with HF compared to patients without HF required inotropes (14/335 (4.2%) vs. 28/3708 (0.8%), *p* < 0.001). No difference was noticed in the use of pressors (74/335 (22.1%) vs. 707/3708 (19.1%), *p* = 0.18), antibiotics other than azithromycin (262/335 (78.2%) vs. 2815/3708 (75.9%), *p* = 0.346), and intravenous steroids (78/335 (23.3%) vs. 819/3708 (22.1%), *p* = 0.614) between the two groups (Table 1).

### 3.4. In-House Mortality and HF

**Figure 1:**
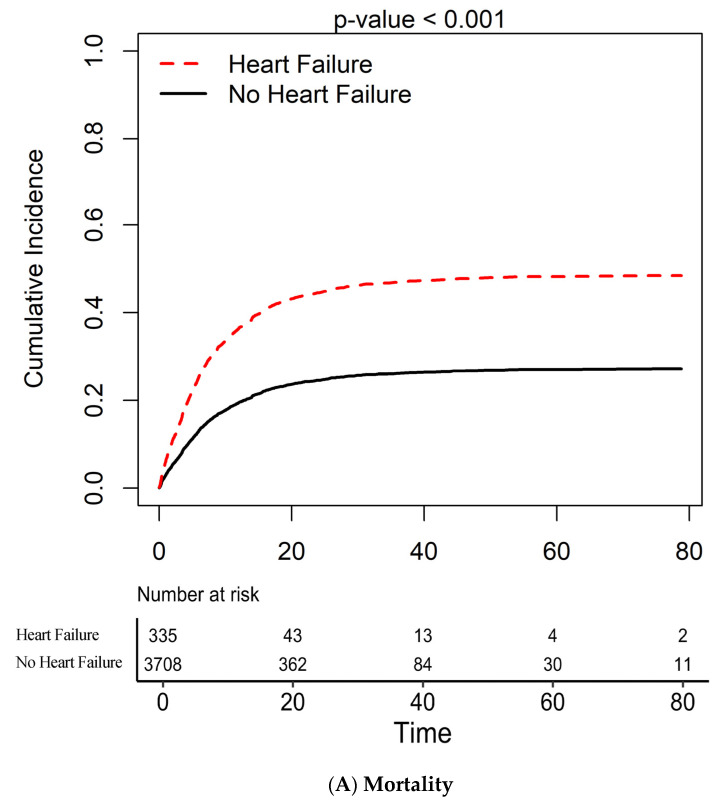

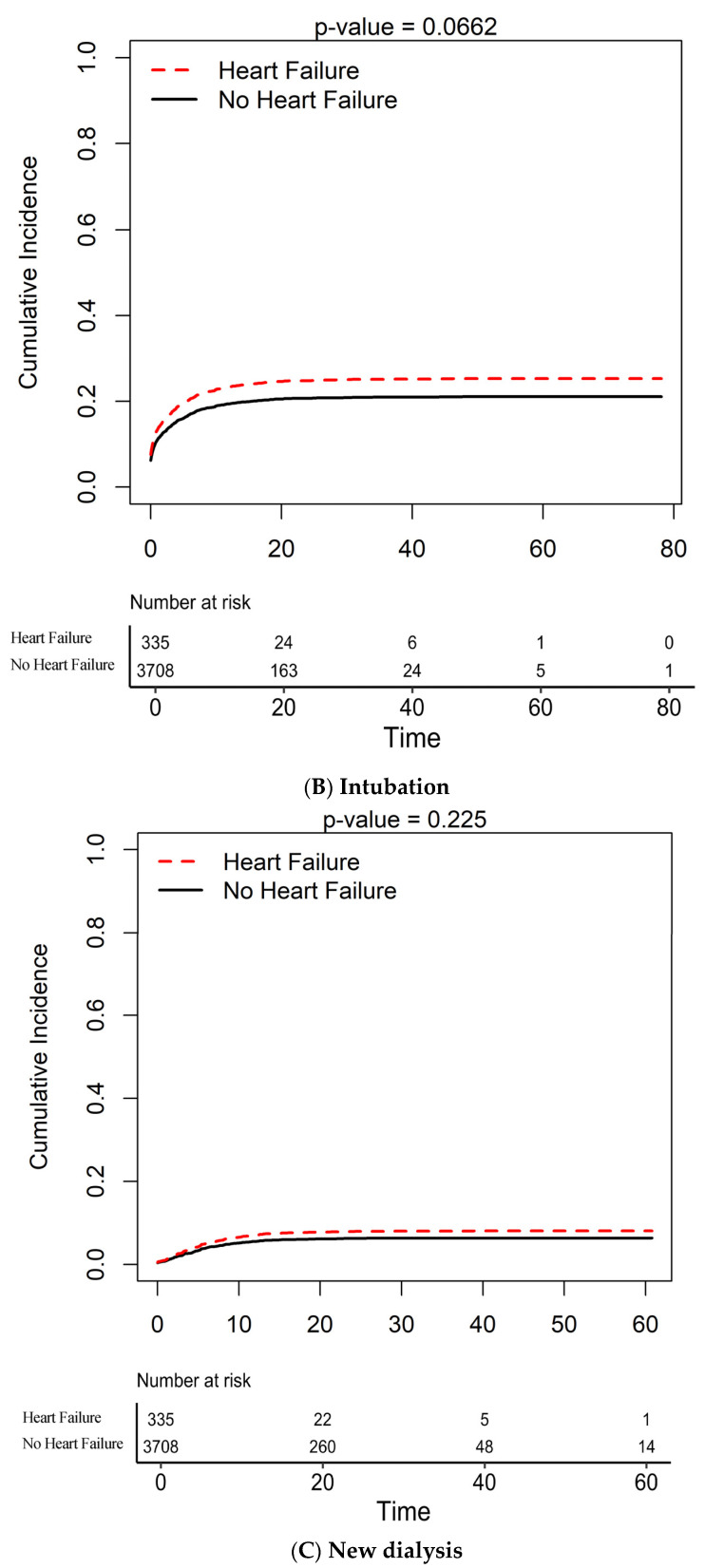
Cumulative incidence of in-hospital (**A**) mortality, (**B**) need for intubation, (**C**) need for intubation in heart failure and non-heart failure patients.

Patients with HF had a higher cumulative incidence of in-hospital mortality compared to patients without HF (164/335 (49.0%) vs. 1007/3708 (27.2%), unadjusted HR = 2.089, *p* < 0.001, Figure 1A). The increased mortality in patients with HF remained statistically significant (HR = 1.383, *p* = 0.001) after adjustment for age, BMI, history of CAD, HTN, COPD, and DM. Propensity score matching confirmed a statistically significant increased risk of in-house mortality for patients with HF (HR = 1.528, *p* = 0.001). Results of propensity score matching are presented in Supplementary Table S2 and reductions in standardized average differences among covariates are shown in Supplementary Figure S1. No difference in in-hospital mortality was observed based on using LVEF as a continuous variable (HR = 0.999, *p* = 0.8) or based on ischemic etiology of HF (HR = 1.368, *p* = 0.11) There was no difference in the cumulative incidence of in-hospital mortality between HFrEF and HFpEF patients. (53/114 (46.5%) vs. 111/221 (50.2%), HR = 0.893, *p* = 0.5, Supplementary Figure S2A).

### 3.5. Need for Intubation and HF

Patients with HF had a trend towards higher cumulative risk of intubation and mechanical ventilation compared to patients without HF (86/335 (25.7%) vs. 789/3708 (21.3%), unadjusted HR = 1.231, *p* = 0.062, Figure 1B), but this was no longer present after adjusting for age, BMI, history of CAD, HTN, COPD, and DM (HR = 1.076, *p* = 0.550), or after propensity score matching (HR = 1.075, *p* = 0.64). There was no significant difference in the cumulative risk of intubation between HFrEF and HFpEF patients (24/114 (21.1%) vs. 62/221 (28.1%), HR = 0.724, *p* = 0.18, Supplementary Figure S2B). No difference in the need for intubation was observed based on using LVEF as a continuous variable (HR = 1.012, *p* = 0.13) or on the ischemic etiology of HF (HR = 1.133, *p* = 0.65).

### 3.6. Need for New Dialysis and HF

There was no difference in the cumulative risk of new dialysis between patients with and without HF (27/335 (7.5%) vs. 237/3708 (6.4%), unadjusted HR = 1.285, *p* = 0.225, Figure 1C). Similar results were observed after adjustment for EGFR at presentation, age, BMI, history of CAD, HTN, COPD, and DM (HR = 1.070, *p* = 0.77) or with propensity score matching (HR = 1.008, *p* = 0.980). There was no difference in the cumulative risk of new dialysis between HFrEF and HFpEF patients (10/114 (8.8%) vs. 16/221 (7.2%) HR = 1.144, *p* = 0.74, Supplementary Figure S2C). No difference in the need for new dialysis was observed based on using LVEF as a continuous variable (HR = 1.007, *p* = 0.6) or on the ischemic etiology of HF (HR = 0.676, *p* = 0.5)

## 4. Discussion

In the present study, we examined the association between HF and in-hospital mortality, need for mechanical ventilatory support, and need for new dialysis in patients hospitalized with COVID-19. Our principal findings are as follows: first, crude mortality in HF patients admitted with COVID-19 was extraordinarily high, with one of two admitted patients dying in-hospital. Second, we identified HF as an independent risk factor for death from COVID-19 with a 50% increase in hazard after propensity matching and controlling for established risk factors including cardiovascular disease such as CAD and hypertension. Lastly, this increased risk is conferred by both HFrEF and HFpEF.

Although the presence of HF in the past medical history has been identified in univariate analysis as one of the risk factors for adverse outcomes in COVID-19 patients [6,7,8,9,14,15,16,17], no prior studies have directly and specifically characterized its independent effect on COVID-19 morbidity (including the need for intubation, dialysis) and mortality. Studies thus far have typically included HF under the umbrella of cardiovascular diseases [6,7,14], or examined composite outcomes [8,15], or built prediction models without adjusting for comorbidities [16,17]. Furthermore, the differences between HFrEF and HFpEF patients in the setting of COVID-19 have never been evaluated. As such, one may consider our large-scale observation novel.

### 4.1. Baseline and Clinical Presentation

HF is seldom an isolated condition, but more often it is associated with different comorbidities [18]. Not surprisingly, the HF patients in our cohort had a higher prevalence of DM, HTN, CAD, and Asthma/COPD when compared to patients without HF. However, both groups presented to the hospital in similar condition, that is, there were no clinically meaningful differences in temperature, systolic blood pressure, pulse oximeter saturation, or respiratory rate on arrival. Notably however, patients with HF had a lower heart rate, likely due to the effect of beta-blockade. Markers of systemic inflammation and elements associated with severity of COVID-19 infection [2,19,20,21,22,23] were also similar between the two groups. The two groups had comparable absolute lymphocyte counts and levels of lactic acid, C-reactive protein, ferritin, AST, and particularly LDH, which has been considered a strong prognostic marker [24]. As expected, pro-BNP was markedly elevated in patients with HF. Not surprisingly given the higher proportion of patients with CAD, median troponin T was also higher in the HF group—although below the threshold to be considered clinically significant (0.09 mg/mL in our institution). Mild differences in D-dimer, hemoglobin level, and worse renal function were likewise observed in the HF groups. 

### 4.2. In-House Mortality

Almost half of the HF patients died during the hospitalization. HF patients were generally older and with more complex medical history. However, the strong association between the history HF and in-house mortality remained significant even after multi-regression adjustment and propensity score matching, proving that the presence of HF is an independent risk factor for in-hospital mortality, irrespective of age and comorbidities.

Taken together, our data demonstrate that even with comparable illness severity at the time of presentation to the hospital, a history of HF is associated with worse outcomes, thereby suggesting that HF itself increases the risk of clinical deterioration during COVID-19 infection.

### 4.3. Intubation, New Hemodialysis, and HF

We observed a marginal increase in the need for mechanical ventilatory support in the HF group that was no longer present after multivariable adjustments or propensity score matching, suggesting that the need for intubation was driven primarily by age, BMI, and other comorbidities than by underlying HF. Similarly, the presence of HF did not increase the need for new hemodialysis. 

### 4.4. HFpEF vs. HFrEF

HFpEF and HFrEF patients had a comparable level of illness at presentation, treatments, and outcomes during their admissions. Although some trends were observed (higher intubation or risk of new hemodialysis in HFpEF patients), none reached statistical significance.

### 4.5. Limitations

Certain limitations of our study should be acknowledged: our data set has the usual limitations of a retrospective chart review analysis with the additional caveat that documentation during the evolving epidemic may have not met usual standards. As an example, NYHA class at presentation was not commonly available. Additionally, this study focused on patients admitted during the initial phase of COVID-19 pandemic, when optimal treatments were unknown, vaccines were not available, and our hospital system was overwhelmed. Mortality rates among HF patients may not be as high with appropriate COVID-19 treatments and more available resources. In particular, the use of remdesivir and dexamethasone, the effects of which became more evident after the initial phase of the pandemic, may have improved mortality among the subset of patients with HF. Lastly, the appearance of COVID-19 mutant strains, not present at the moment of this study, may differently affect the outcome patients with HF.

Heart failure represents an independent risk factor for mortality in patients admitted with COVID-19 infection. As treatment algorithms for COVID-19 evolve, the presence of underlying HF should be taken into consideration when assessing the risk/benefit ratios of aggressive early intervention, including the prioritization of vaccination for patients with HF.

## Figures and Tables

**Table 1:** Characteristics of patients admitted with COVID-19.

	Without Heart Failure	With Heart Failure	*p*
	*n* = 3708	*n* = 335	
Demographics			
Age, year	65 (54–76)	73 (65–82)	<0.001
Male gender, no (%)	1925/3708 (51.9)	190/335 (56.7)	0.092
BMI (kg/m^2^)	28.8 (24.9–33.3)	27.9 (24.1–32.9)	0.063
Past Medical History			
Diabetes, no (%)	1827/3708 (49.3)	247/335 (73.7)	<0.001
Hypertension, no (%)	2577/3708 (69.5)	308/335 (91.9)	<0.001
CAD, no (%)	723/3708 (19.5)	249/335 (74.3)	<0.001
Asthma/COPD, no (%)	939/3708 (25.3)	176/335 (52.5)	<0.001
Presentation			
Symptom duration, days	3 (0–7)	2 (0–7)	0.265
Temperature, F	98.9 (98.2–100.1)	98.7 (97.9–99.9)	0.002
Systolic BP, mmHg	130 (114–147)	130.5 (113–147)	0.737
Diastolic BP, mmHg	75 (65–84)	73 (60–82)	0.001
HR, bpm	99 (87–113)	92 (78–106)	<0.001
Pulse oximeter saturation, %	95 (90–98)	95 (91–98)	0.047
Respiratory rate, bpm	20 (18–22)	20 (18–24)	0.274
WBC count, k/µL	7.6 (5.6–10.5)	7.4 (5.3–10.4)	0.293
Lymphocytes count, k/µL	1 (0.7–1.4)	1 (0.7–1.4)	0.123
Hemoglobin, g/dL	13 (11.6–14.3)	11.9 (10.2–13.6)	<0.001
Platelet count, k/µL	190 (119–259)	169.5 (112.3–242.8)	0.049
Sodium, mEq/L	137 (134–141)	137 (134–142)	0.762
Potassium, mEq/L	4.3 (3.9–4.7)	4.6 (4.1–5)	<0.001
Chloride, mEq/L	99 (95–103)	99 (95–103)	0.455
Bicarbonate, mEq/L	25 (22–27)	25 (21–28)	0.07
EGFR, mL/min/BSA	66 (40.5–89)	40.7 (22.8–59.2)	<0.001
AST, U/L	42 (28–67)	36 (25–57)	0.001
ALT, U/L	28 (18–46)	22 (14–36)	<0.001
Lactic acid, mmol/L	2.1 (1.6–3)	2.3 (1.6–3.2)	0.161
Creatinine Kinase, U/L	182 (90–451)	151 (76–342)	0.008
ProBNP, pg/mL	306 (90–1096)	2727 (1068–9108)	<0.001
D-dimer, µg/mL	1.7 (0.9–4)	2.5 (1.1–5.4)	0.024
C-reactive protein, µg/mL	10.6 (4.4–18.4)	10 (4.1–21.8)	0.759
LDH, U/L	401 (294–549)	392 (284–562)	0.846
Ferritin, ng/mL	739 (357–1472)	615 (323–1155)	0.065
IL6, pg/mL	33.2 (14.5–74.9)	41.1 (18.9–102.8)	0.06
Procalcitonin, ng/mL	0.2 (0.1–0.7)	0.3 (0.1–1)	0.024
Troponin T, ng/mL	0.01 (0.01–0.02)	0.03 (0.01–0.07)	<0.001
Treatments during admission			
Beta Blocker	920/3708 (24.8)	205/335 (61.2)	<0.001
ACE-I	259/3708 (7.0)	41/335 (12.5)	<0.001
ARBs	230/3708 (6.2)	22/335 (6.6)	0.792
Pressors, no (%)	707/3708 (19.1)	74/335 (22.1)	0.180
Inotropes, no (%)	28/3708 (0.8)	14/335 (4.2)	<0.001
Hydroxychloroquine, no (%)	2645/3708 (71.3)	203/335 (60.6)	<0.001
Chloroquine, no (%)	58/3708 (1.6)	6/335 (1.8)	0.75
Azithromycin, no (%)	1205/3708 (32.5)	84/335 (25.1)	0.005
Other antibiotics, no (%)	2815/3708 (75.9)	262/335 (78.2)	0.346
IV steroids, no (%)	819/3708 (22.1)	78/335 (23.3)	0.614

ACE-I = Angiotensin converting enzyme inhibitor; ALT = Alanine transaminase; ARBs = Angiotensin II receptor blocker; AST = Aspartate transaminase; BMI = Body mass index; BP = Blood pressure; CAD = Coronary artery disease; COPD = Chronic obstructive pulmonary disease; EGFR = Estimated glomerular filtration rate; F = Fahrenheit; G6PD = Glucose-6-phosphate dehydrogenase deficiency; HR = Heart rate; IL-6 = Interleukin 6; IV = Intravenous; LDH = Lactate dehydrogenase; proBNP = ProB-type Natriuretic Peptide; WBC = White blood cell.

## Data Availability

The data presented in this study are available on request from the corresponding author. The data are not publicly available due to ongoing clinical investigations.

## Supplementary Materials

The following are available online at https://www.mdpi.com/article/10.3390/jcdd8070077/s1. Supplementary Figure S1. Standardized differences of covariates before and after propensity matching score. Supplementary Figure S2. Cumulative incidence of in-hospital (**A**) mortality, (**B**) need for intubation, (**C**) need for intubation in heart failure with reduced ejection fraction (HFrEF) and heart failure with preserved ejection fraction (HFpEF) patients. Supplementary Table S1, Characteristics of heart failure patients admitted with COVID-19. Supplementary Table S2. Demographics and past medical history of propensity matched patients.
