# Supplementary material for: A History of Heart Failure Is an Independent Risk Factor for Death in Patients Admitted with Coronavirus 19 Disease
Source: J Cardiovasc Dev Dis. 2021 Jun 30;8(7):77. doi: 10.3390/jcdd8070077 (PMC8307512; doi:10.3390/jcdd8070077)
Supplement: Supplementary file 1 [file jcdd-08-00077-s001.zip › jcdd-1269193-SI.pdf]

Supplemental Table I. Characteristics of heart failure patients admitted with COVID-19

	HFpEF	HFrEF	p
	**n=221**	**n=114**	
**Demographics**			
Age, year	74 (65 - 83)	73 (65.8 - 79.3)	0.256
Male gender, no (%)	113/221 (51.1)	77/114 (67.5)	0.004
BMI (kg/m^2)	28.4 (24.6 - 33.8)	26.7 (23 - 31.2)	0.017
**Past Medical History**			
Diabetes, no (%)	162/221 (73.3)	85/114 (74.6)	0.804
Hypertension, no (%)	203/221 (91.9)	105/114 (92.1)	0.937
CAD, no (%)	158/221 (71.5)	91/114 (79.8)	0.098
Asthma/COPD, no (%)	130/221 (58.8)	46/114 (40.4)	0.001
**Presentation**			
Symptom duration, days	2 (0 - 7)	2 (0 - 7)	0.294
Temperature, F	98.8 (98 - 100)	98.5 (97.8 - 99.7)	0.041
Systolic BP, mmHg	134 (116 - 149)	127 (104 - 144)	0.015
Diastolic BP, mmHg	73 (61 - 82)	72 (58.5 - 81.5)	0.757
HR, bpm	93 (80 - 106.5)	90 (74 - 106)	0.129
Pulse oximeter saturation, %	95 (91 - 98)	96 (92 - 99)	0.187
Respiratory rate, bpm	20 (18 - 24)	20 (18 - 24)	0.425
WBC count, k/μL	7.3 (5.3 - 10.6)	7.9 (5.4 - 10.2)	0.662
Lymphocytes count, k/μL	1 (0.7 - 1.3)	1 (0.6 - 1.4)	0.8
Hemoglobin, g/dL	12.1 (10.2 - 13.9)	11.6 (10.2 - 13.4)	0.319
Platelet count, k/μL	170 (118 - 242.5)	169 (91 - 244)	0.83
Sodium, mEq/L	137 (133 - 142)	138 (135 - 141)	0.845
Potassium, mEq/L	4.6 (4.1 - 4.9)	4.6 (4.2 - 5.1)	0.397
Chloride, mEq/L	99 (95 - 104)	99 (95 - 103)	0.68
Bicarbonate, mEq/L	25 (21 - 28)	24 (21 - 27)	0.403
EGFR, mL/min/BSA	40.9 (23.7 - 62.5)	40.2 (20.1 - 56.9)	0.275
AST, U/L	36 (25.3 - 56)	36 (24 - 61.5)	0.857
ALT, U/L	22 (14 - 36)	25 (14 - 35.5)	0.369
Lactic acid, mmol/L	2.2 (1.6 - 2.9)	2.4 (1.7 - 3.7)	-0.044
Creatinine Kinase, U/L	151 (74.8 - 334.3)	148 (77.3 - 366.8)	0.919
ProBNP, pg/mL	2328 (877.5 - 5411.5)	5416 (1371.5 - 15000)	0.001
D-dimer, μg/mL	2.1 (1.1 - 5)	3.3 (1.4 - 7.6)	0.19
C-reactive protein, μg/mL	10 (3.5 - 21.3)	10.3 (6 - 22.3)	0.397
LDH, U/L	379 (280 - 556)	412 (303 - 570)	0.415
Ferritin, ng/mL	620 (329.3 - 1308)	593 (241 - 1027.8)	0.449
IL6, pg/mL	42.3 (18.9 - 110.5)	39 (17.7 - 89.1)	0.624
Procalcitonin, ng/mL	0.3 (0.1 - 0.9)	0.4 (0.1 - 1.6)	0.729
Troponin T, ng/mL	0.02 (0.01 - 0.06)	0.04 (0.01 - 0.1)	0.006
**Treatments during admission**			
Beta Blocker	129/221 (58.4)	76/114 (66.7)	0.140
ACE-i	22/221 (10.0)	19/114 (16.7)	0.076
ARBs	15/221 (6.8)	7/114 (6.1)	0.821
Pressors, no (%)	49/221 (22.2)	25/114 (21.9)	0.960
Inotropes, no (%)	3/221 (1.4)	11/114 (9.6)	0.000
Hydroxychloroquine, no (%)	141/221 (63.8)	62/114 (54.4)	0.095
Chloroquine, no (%)	3/221 (1.4)	3/114 (2.6)	0.405
Azithromycin, no (%)	59/221 (26.7)	25/114 (21.9)	0.34
Other antibiotics, no (%)	172/221 (77.8)	90/114 (78.9)	0.814
IV steroids, no (%)	61/221 (27.6)	17/114 (14.9)	0.009

ACE-I = Angiotensin converting enzyme inhibitor; ALT= Alanine transaminase; ARBs = Angiotensin II receptor blocker; BMI = Body mass index; BP = Blood pressure; CAD = Coronary artery disease; COPD = Chronic obstructive pulmonary disease; EGFR = Estimated glomerular filtration rate; F= Fahrenheit; G6PD = Glucose-6-phosphate dehydrogenase deficiency; HFpEF = Heart failure with preserved ejection fraction; HFrEF = Heart failure with reduced ejection fraction; HR = Heart rate; IL-6 = Interleukin 6; IV= Intravenous; LDH = Lactate dehydrogenase; proBNP = ProB-type Natriuretic Peptide; WBC = White blood cell.

Supplemental Table II. Demographics and past medical history of propensity matched patients

	**Without Heart Failure**	**With Heart Failure**	**p**
	n=328	n=328	
**Demographics**			
Age, year	73 (65 - 80)	73 (65 - 81)	0.36
BMI (kg/m^2)	28.8 (25 - 33)	28 (24.1 - 33)	0.39
**Past Medical History**			
Diabetes, no (%)	250/328 (76.2)	243/328 (74.1)	0.53
Hypertension, no (%)	301/328 (91.8)	302/328 (92.1)	0.89
CAD, no (%)	243/328 (74.1)	244/328 (74.4)	0.93
Asthma/COPD, no (%)	175/328 (53.4)	173/328 (52.7)	0.88

BMI = Body mass index; CAD = Coronary artery disease; COPD = Chronic obstructive pulmonary disease.

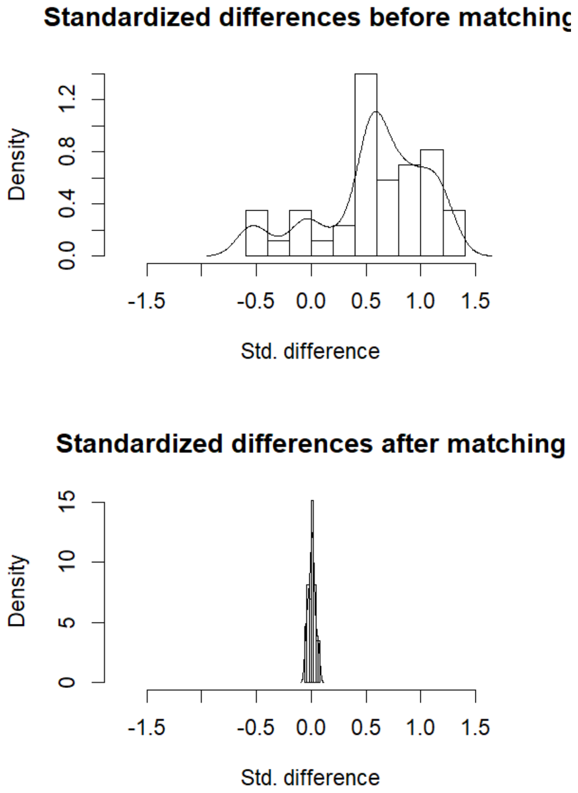

Supplemental Figure I. Standardized differences of covariates before and after propensity matching score.

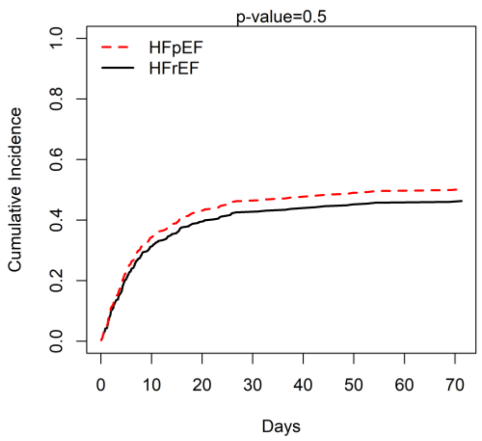

(A) Mortality

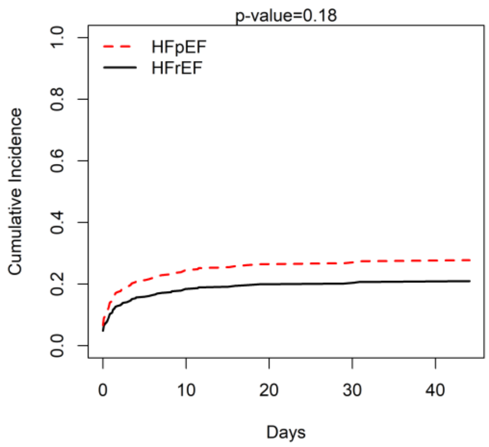

(B) Intubation

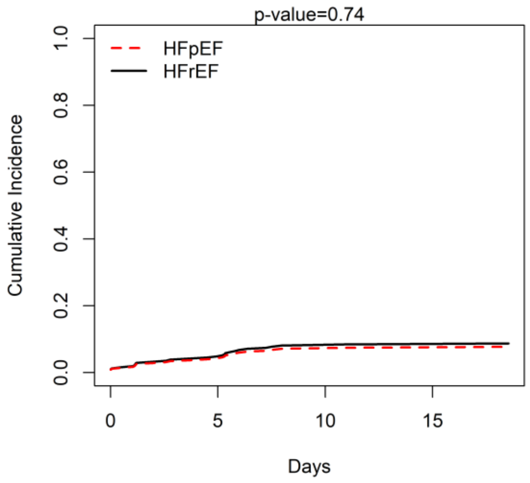

(C) New Dialysis

Supplemental Figure II. Cumulative incidence of in-hospital (A) mortality, (B) need for intubation, (C) need for intubation in heart failure with reduced ejection fraction (HFrEF) and heart failure with preserved ejection fraction (HFpEF) patients.
